# Employing Copper-Based Nanomaterials to Combat Multi-Drug-Resistant Bacteria

**DOI:** 10.3390/microorganisms13040708

**Published:** 2025-03-21

**Authors:** Yujie Zhai, Zhuxiao Liang, Xijun Liu, Weiqing Zhang

**Affiliations:** 1 State Key Laboratory of Featured Metal Materials and Life-Cycle Safety for Composite Structures, School of Resources, Environment and Materials, Guangxi University, Nanning 530004, China; zyj_000831@163.com; 2 Department of Research, Guangxi Medical University Cancer Hospital, Nanning 530021, China; lzzxx13@163.com

**Keywords:** drug-resistant bacteria, antibacterial mechanism, Cu-based nanomaterials

## Abstract

The rise of multi-drug-resistant (MDR) bacteria poses a severe global threat to public health, necessitating the development of innovative therapeutic strategies to overcome these challenges. Copper-based nanomaterials have emerged as promising agents due to their intrinsic antibacterial properties, cost-effectiveness, and adaptability for multifunctional therapeutic approaches. These materials exhibit exceptional potential in advanced antibacterial therapies, including chemodynamic therapy (CDT), photothermal therapy (PTT), and photodynamic therapy (PDT). Their unique physicochemical properties, such as controlled ion release, reactive oxygen species (ROS) generation, and tunable catalytic activity, enable them to target MDR bacteria effectively while minimizing off-target effects. This paper systematically reviews the mechanisms through which Cu-based nanomaterials enhance antibacterial efficiency and emphasizes their specific performance in the antibacterial field. Key factors influencing their antibacterial properties—such as electronic interactions, photothermal characteristics, size effects, ligand effects, single-atom doping, and geometric configurations—are analyzed in depth. By uncovering the potential of copper-based nanomaterials, this work aims to inspire innovative approaches that improve patient outcomes, reduce the burden of bacterial infections, and enhance global public health initiatives.

## 1. Introduction

Antibiotic resistance has emerged as a global health crisis, posing significant challenges to modern medicine and scientific research [1,2]. The increasing prevalence of bacterial infections, coupled with the declining efficacy of traditional antibiotics, has led to an alarming surge in antibiotic-resistant strains. The misuse and overuse of antibiotics, once the mainstay of treatment for bacterial infections, have accelerated the emergence of multi-drug-resistant pathogens or “superbugs”. These pathogens are resistant to multiple classes of antibiotics, rendering conventional treatments ineffective and resulting in untreatable infections, increased healthcare costs, and escalating mortality rates [3,4,5]. With once-reliable antibiotics losing their potency, the urgent need for alternative antibacterial strategies has never been more critical [6,7].

In response to this escalating threat, nanomaterials have emerged as promising candidates in the next generation of antibacterial therapies. Unlike traditional antibiotics, nanomaterials provide distinct advantages through their multifaceted mechanisms of action, particularly against drug-resistant bacterial infections [8,9,10] (Table 1). Among the various nanomaterials under investigation, copper-based nanomaterials have garnered significant attention due to their broad-spectrum antibacterial activity [11,12,13]. These materials mitigate the risk of resistance development by targeting bacteria through multiple pathways, including the disruption of cell membranes, the generation of reactive oxygen species (ROS), and interference with vital enzyme functions. Specifically, they disrupt bacterial cell membranes, generate reactive oxygen species, and interfere with critical enzyme functions. This multipronged strategy positions copper-based nanomaterials as a powerful and versatile solution to the growing challenge of antibiotic resistance [14,15,16].

**Figure 1:**
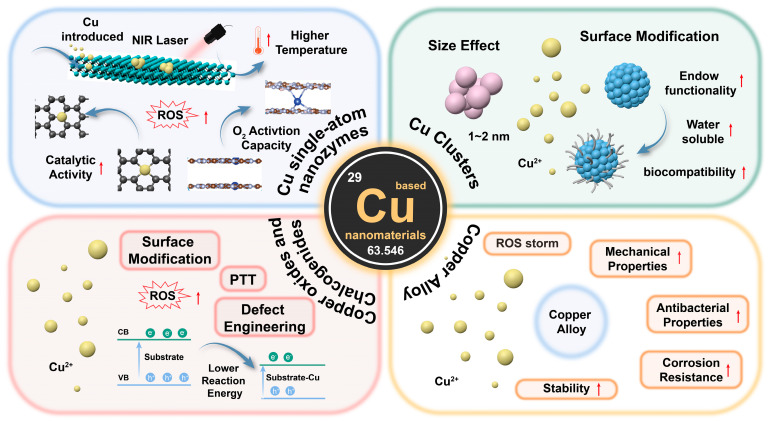
Schematic illustration of antibacterial therapy applications based on construction of copper nanostructured materials. The red arrows represent an upward trend.

In recent years, copper-based nanomaterials have been widely explored not only for antibacterial applications but also in tumor therapies (Figure 1) [33,34,35]. Despite the wide range of applications of copper nanomaterials in various fields in dental water treatment [36,37,38], there are still a limited number of relevant reviews on how to optimize the antibacterial efficiency of copper nanomaterials. Furthermore, emerging antibacterial strategies utilizing copper-based nanomaterials, such as chemodynamic therapy (CDT) [39,40], photothermal therapy (PTT) [41,42], and photodynamic therapy (PDT) [43], offer new opportunities to combat resistant pathogens. These advancements underscore the critical need to comprehensively summarize recent advances in copper-based nanomaterials for antibacterial purposes.

This review aims to address this gap by focusing on the mechanisms through which copper-based materials enhance antibacterial efficiency, their diverse applications in combating bacterial infections, and, more specifically, the role of copper oxide and copper sulfide in antibacterial therapy. Additionally, we delve into key factors influencing the antibacterial performance of these materials, including electronic interactions, photothermal properties, size effects, ligand modifications, single-atom doping, and geometric configurations. By unlocking the potential of copper-based nanomaterials, this review seeks to expand their applications in addressing drug-resistant pathogens, ultimately improving patient outcomes and advancing global public health.

## 2. Key Antibacterial Targets: Membrane Integrity, ROS, Metabolism/Enzymes, DNA, and Signaling Pathways

**Figure 2:**
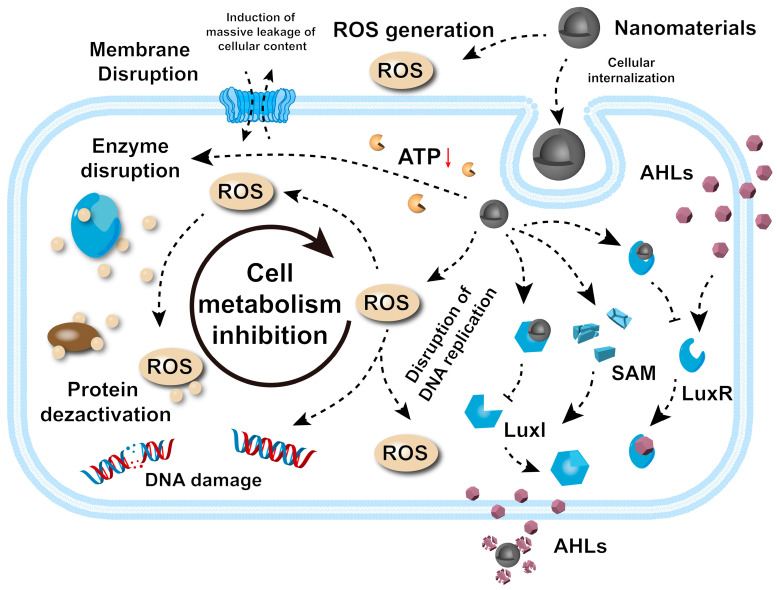
Antibacterial mechanisms behind nanomaterials. The red arrow represents a downward trend.

Understanding the pathways through which nanomaterials exert their antibacterial effects is essential in the rational design and development of next-generation therapeutic agents. These mechanisms can be categorized into five principal pathways: the disruption of bacterial cell membrane integrity, the generation of reactive oxygen species (ROS), interference with bacterial metabolism and enzyme function, DNA damage, and disruption to bacterial signaling pathways. Each pathway induces specific biochemical and structural disruptions, inhibiting bacterial proliferation or causing cell death (Figure 2).

### 2.1. Membrane Disruption

The destruction of bacterial membranes by nanomaterials represents a key antibacterial mechanism. The process is initiated by electrostatic interactions between positively charged nanoparticles and negatively charged components of the bacterial membrane, such as phospholipids and lipopolysaccharides in Gram-negative bacteria. These interactions bring nanoparticles into close proximity with the cell membrane, enabling further interactions. Upon contact, nanoparticles can penetrate the lipid bilayer, causing physical damage such as puncturing or thinning and compromising membrane integrity [44,45,46]. This leads to an uncontrolled flow of ions and molecules across the membrane, disrupting cellular homeostasis and function. In addition, nanoparticles may interact with and denature key membrane proteins, further exacerbating membrane instability [47]. These combined effects result in membrane collapse, the leakage of intracellular components (e.g., DNA, RNA, proteins), and eventual bacterial death. This mechanism is particularly effective against drug-resistant strains, positioning nanomaterials as potent alternatives to traditional antibiotics.

Notably, Stefan Howorka’s team has pioneered the development of cholesterol-modified DNA nanostructures, negatively charged nanostructures that preferentially bind to cholesterol-deficient bacterial membranes and attach to the membrane surface by aggregating to form barrel-like clusters, which triggers the rupture of the membrane, leading to cell death [48]. This selective disruption of bacterial membranes provides an idea for the development of novel antimicrobial agents, especially for antibiotic resistance, and small molecule or polymer drugs may be designed in the future by mimicking this principle.

### 2.2. Generation of Reactive Oxygen Species (ROS)

ROS have attracted attention for their broad-spectrum, efficient, and long-lasting bactericidal effects. ROS, including superoxide anions (O_2_^−^), hydroxyl radicals (·OH), and hydrogen peroxide (H_2_O_2_), are highly reactive molecules capable of inflicting extensive oxidative damage on bacterial cells, ultimately leading to cell death [49,50]. Nanomaterials facilitate ROS generation through several mechanisms. For example, titanium dioxide (TiO_2_) nanoparticles, when activated by UV light, form electron–hole pairs that catalyze surface redox reactions to produce ROS [51,52,53]. Similarly, silver nanoparticles (Ag) promote electron transfer from bacterial membrane components to molecular oxygen, generating O_2_^−^ and disrupting bacterial respiration [54,55]. Iron oxide nanoparticles (Fe_3_O_4_) participate in Fenton and Fenton-like reactions, where Fe^2+^ reacts with H_2_O_2_ to generate ·OH, one of the most bactericidal potent ROS [56,57,58]. The resulting ROS damage bacterial DNA, proteins, and lipids. Lipid peroxidation impairs membrane integrity, increasing permeability and causing the leakage of bacterial contents [59]. ROS-induced DNA damage, including strand breaks and base modifications, disrupts replication and transcription, further inhibiting bacterial survival.

Yu et al. have developed an ROS nanogenerator (Fe-HMME@DHA@MPN) activated by ultrasound and gastric acid, which can effectively kill drug-resistant bacteria [60]. The nanogenerator utilizes the Fenton reaction to generate ROS and self-supplies hydrogen peroxide through encapsulated DHA to ensure the continuous generation of ROS in the absence of exogenous H_2_O_2_. The antibacterial mechanism and adhesion properties of ROS also make it highly capable of destroying the biofilm, which in turn kills drug-resistant *H. pylori* and destroys its biofilm. These multifaceted mechanisms make ROS-generating nanomaterials highly effective against antibiotic-resistant strains, highlighting their potential in antibacterial therapies.

### 2.3. Interference with Bacterial Metabolism and Enzyme Function

Nanomaterials can disrupt bacterial metabolic processes and enzymatic functions, primarily through metal-catalyzed oxidation and metal ion toxicity. In *E. coli*, oxidative damage often targets specific amino acids near metal-binding sites, leading to protein carbonylation and degradation [61,62]. Metalloenzymes, such as peptide deformylase (PDF) and threonine dehydrogenase (Tdh), are particularly susceptible to oxidative damage catalyzed by Fe^2+^, though alternative metals such as Mn^2+^ can mitigate this damage [63]. In addition, toxic metal ions, such as Cu^+^, disrupt Fe-S cluster-containing enzymes essential in bacterial metabolism, inhibiting their activity and bacterial growth [64]. Other ions, including Ag^+^ and Cd^2+^, inactivate Fe-S enzymes, though bacterial repair mechanisms can sometimes restore their function [65]. Metal substitution, such as Pb^2+^ replacing Zn^2+^ in δ-aminolaevulinic acid dehydrogenase (ALAD) or Ag^+^ replacing Cu^2+^ in superoxide dismutase, also inhibits enzyme function, preventing bacterial proliferation [66,67].

Furthermore, the researchers developed a gastric acid-responsive nanocarrier, CAMO, which inhibited Helicobacter pylori urease activity through the acid-triggered release of silver ions (Ag^+^), reduced bacterial resistance, and synergistically enhanced the antibacterial effect of clarithromycin [30]. At the same time, the outer probiotic layer reduces inflammation by targeting and protecting gastric epithelial cells, carrying out the dual mechanism of antibiotic potentiation and host protection, which provides a new strategy for precise delivery and synergistic therapy for the treatment of drug-resistant bacterial infections.

### 2.4. DNA Damage

Nanomaterials exhibit potent antibacterial effects by inducing bacterial DNA damage through direct and indirect mechanisms [68]. For instance, Ag nanomaterials penetrate bacterial cells and bind to DNA, causing structural damage such as strand breaks, cross-linking, and adduct formation, thereby disrupting critical processes such as replication and transcription [69]. Nanomaterials also inhibit DNA repair enzymes, including polymerases, helicases, and ligases, impairing the ability of cells to repair DNA damage and exacerbating its effects [70]. Additionally, E-CuSe inhibits the self-repair ability of bacteria by down-regulating DNA repair-related genes (such as RecA, UvrD, etc.), meaning that bacteria are unable to complete the dynamic repair of DNA damage caused by copper ion attack and eventually die due to the cumulative irreversible damage. This gene regulation mechanism cooperates with the physical effect of E-CuSe to destroy the bacterial cell membrane, breaking through the defense threshold of drug-resistant bacteria and significantly enhancing the antibacterial efficiency [45]. Collectively, these mechanisms make nanoparticles particularly effective against resistant bacteria, offering a robust alternative to conventional antibiotics.

### 2.5. Disruption of Bacterial Signaling (Quorum Sensing System)

Quorum sensing (QS) is a bacterial communication system that regulates physiological processes such as biofilm formation, virulence factor production, motility, and drug resistance mechanisms [71,72]. Nanomaterials have been found to disrupt QS, hindering bacterial collaboration and impairing their ability to coordinate essential functions. These disruptions can be achieved through various mechanisms: (I) the inhibition of signal molecule production (auto-inducible factors)—nanomaterials interfere with bacterial metabolic pathways involved in the synthesis of autoinducer molecules, reducing the overall signaling capacity [73]; (II) signal quenching—nanoparticles bind directly to signaling molecules, such as autoinducers, neutralizing their activity and preventing them from interacting with QS receptors [74,75]; and (III) receptor blocking—nanomaterials can obstruct QS receptors, inhibiting the detection and response to signaling molecules [76]. By disrupting QS, nanomaterials inhibit biofilm formation, reduce virulence factor production, and enhance the susceptibility of bacteria to antibacterial agents.

Notably, such mechanisms have been validated in several studies [77]. For example, researchers synthesized Zn-Nx-C nanomaterials with endostatin-like activity and demonstrated their ability to degrade AHL signaling molecules, interfere with the QS signaling pathway, and inhibit biofilm formation in a multi-drug-resistant *Pseudomonas aeruginosa* model. The Zn-Nx-C coating was also effective in inhibiting biofilm formation on the surface of hull materials in an aqueous environment. After 30 days of exposure in the Suhang Grand Canal, the coating achieved 80.3% biofilm inhibition efficiency. These properties make nanomaterials a promising strategy for combating drug-resistant infections and minimizing bacterial pathogenicity [78].

## 3. Cu-Based Nanomaterials for Biocatalytic Antibacterial Applications

In recent years, copper-based nanomaterials have demonstrated breakthrough research progress in the field of antibacterial therapeutics due to their unique compositional and structural features, providing an important scientific basis for the development of novel antibacterial strategies against drug-resistant bacterial infections [79,80,81]. In this subsection, we will systematically review recent research progress in copper-based antimicrobial nanomaterials with different chemical coordination environments and compositional features, from copper single-atom systems precisely tuned on the atomic scale (Cu-N_3_, Cu/PMCs) to copper nanoclusters with quantum size effects (Cu_8_NC, CuCs) and from redox-active copper oxides (Cu_5.4_O) to narrow bandgap-characterized copper sulfur compounds (CuS, Cu_2−x_Se), as well as other copper-doped nanomaterials. The research focuses on the analysis of the conformational relationships of the nanomaterials and the mechanism behind the antimicrobial effect, which will provide theoretical support for the rational design of new nano-antibacterial agents.

### 3.1. Cu Single-Atom Nanozymes

Single-atom nanomaterials (SACs) represent an innovative class of catalysts where individual metal atoms are dispersed on suitable support materials, maximizing their atomic efficiency and catalytic activity [82,83,84,85]. These cutting-edge materials have gained significant attention for diverse applications. SACs exhibit broad-spectrum antibacterial activity, including potent effects against multi-drug-resistant strains, positioning them as promising candidates to address the escalating issue of antibiotic resistance. They exhibit rapid and effective bactericidal action by disrupting bacterial cell walls, membranes, and genetic materials [41,86,87,88]. SACs are classified based on the metal type and the support structure, which can range from carbon-based materials [89,90,91] to metal oxides [92,93] and other functional nanostructures [94,95]. Among these, Cu SACs hold particular promise due to their remarkable redox activity and photothermal properties.

**Figure 3:**
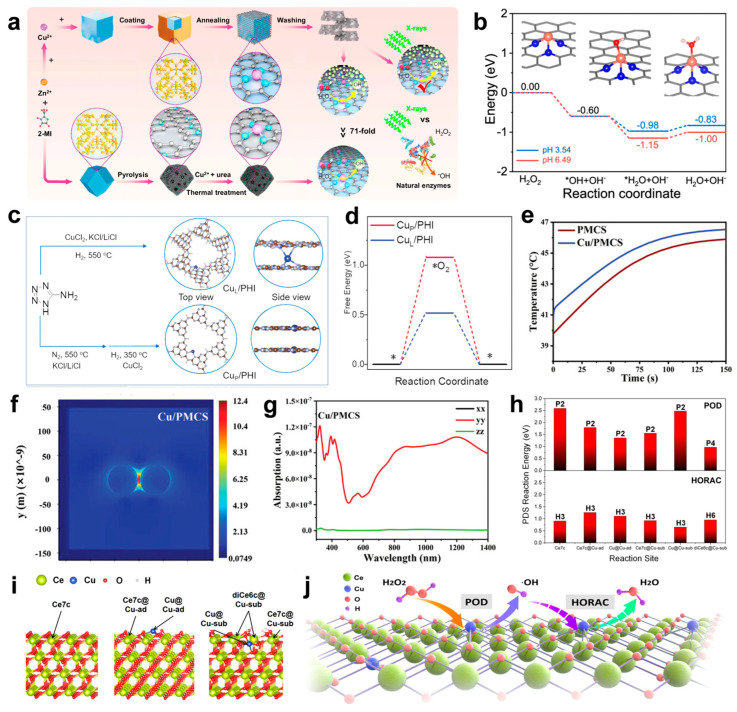
(**a**) Schematic diagram of the synthesis strategy of CuN_3_-SAzyme and CuN_4_-SAzyme. 2-methylimidazole: 2-MI. (**b**) Calculated energy profiles for the dissociation of the H_2_O_2_ molecule on CuN_3_-SAzyme at two experimental pH values. Note that these produced ·OH radicals are very easily involved in reactions around them, which can lead to a further decrease in the energy of the whole system. Cu, orange; C, gray; N, blue; O, red; H, pink. (**c**) Scheme of the synthesis of Cu_L_/PHI and Cu_P_/PHI, N (gray), Cu (blue), H (white), and C (brown). An asterisk (*) represents the surface position of the adsorption. (**d**) DFT calculation of free energy of O_2_ adsorption (ΔG*O_2_) for Cu_P_/PHI (red) and Cu_L_/PHI (blue). (**e**) Heating curve under 808 nm NIR irradiation with a power density of 1 W cm^−2^ for 150 s plotted by simulation. (Cu/PMCS refers to Cu/PMCS-2). (**f**) Thermal field maps of Cu/PMCS plasmons. (**g**) Absorption spectra at 808 nm of Cu/PMCS. (**h**) The calculated PDS reaction energy of POD and HORAC processes for different reaction sites with the exact PDS labeled above the bar. (**i**) The calculated model of pristine CeO_2_ (left), CeO_2_ with Cu3c added on the 3O atoms on the surface (Cu-ad, middle) and CeO_2_ with a Ce atom substituted by Cu (Cu-sub, right). The six possible reaction sites are highlighted with a tagged arrow. (**j**) The proposed mechanism of regulation of catalytic activities by Cu-CeO_2_ single-site nanozyme. (**a**,**b**) Reproduced with permission [96]. Copyright 2024, Nature Publishing Group. (**c**,**d**) Reproduced with permission [88]. Copyright 2023, WILEY-VCH. (**e**–**g**) Reproduced with permission [100]. Copyright 2024, WILEY-VCH. (**h**–**j**) Reproduced with permission [92]. Copyright 2024, Nature Publishing Group.

The rational design of nanozymes hinges on sophisticated strategies, with coordination engineering and spatial configuration optimization emerging as critical approaches to enhancing their catalytic efficiency and antibacterial performance. Coordination engineering, as a pivotal strategy, refines the coordination number and ligand environment around Cu atoms to boost POD-like activity (Figure 3a), facilitating efficient H_2_O_2_ decomposition into ·OH (Figure 3b) [96]. Cu SACs with lower coordination numbers (e.g., CuN_3_) exhibit superior H_2_O_2_ adsorption and dissociation compared to their CuN_4_ counterparts, resulting in enhanced ROS generation and antibacterial activity. Furthermore, tailored coordination structures significantly improve the structural stability and catalytic performance of SACs, ensuring adaptability to diverse reaction conditions, such as variations in energy radiation, pH, and temperature. Beyond coordination optimization, the spatial modulation of Cu atom positioning further elevates catalytic performance [88]. A recent study systematically evaluated Cu SACs with dual Cu sites on poly (heptazine imide) (PHI) nanoplatforms, comparing interlayer (Cu_L_/PHI) and in-plane (Cu_P_/PHI) configurations (Figure 3c). The results demonstrated that interlayer localization (Cu_L_/PHI) notably enhances photoinduced electron transfer, O_2_ activation (Figure 3d), and ROS generation, achieving near-complete bactericidal activity under visible light. Notably, SACs with interlayer Cu_L_/PHI exhibited superior antibacterial efficacy and stability over their in-plane counterparts. These findings underscore the synergistic roles of coordination engineering and atomic spatial configuration in advancing the rational design of nanozymes, offering valuable insights for biomedical applications.

Building on these advancements, recent studies have highlighted the potential of Cu doping to enhance the photothermal properties of nanomaterials, opening up new avenues for performance optimization [97,98,99]. Liu et al. demonstrated that Cu doping significantly amplifies the photothermal effect of PMCS by introducing spin–orbit coupling and d-orbital transitions (Figure 3e) [100]. These transitions facilitate energy release via inter-system crossing rather than emission. Additionally, Cu doping generates impurity energy levels that enhance dielectrophoretic absorption and induce localized surface plasmon resonance (LSPR). As a result, Cu/PMCS exhibits plasmon generation at 808 nm, a feature absent in pure PMCS (Figure 3f). Simulations further confirmed that Cu/PMCS achieves notable absorption and higher thermal output at 808 nm, attributed to the LSPR effect (Figure 3g).

Following the progress made with carbon-based copper single-atom catalysts, attention has turned to metal oxide-supported systems, such as copper anchored on ceria, which exhibit distinct advantages in catalytic and antibacterial applications [93,101,102]. Anchoring copper onto CeO_2_ enhances POD-like activity, as elucidated by theoretical calculations (Figure 3h,i), which reveal the mechanism behind Cu single sites promoting ROS generation [92]. Specifically, the Cu-sub configuration induces structural modifications that activate adjacent Ce sites by altering local coordination and oxygen alkalinity, thereby stabilizing reaction intermediates. Energy analyses indicate that Cu single sites markedly lower the potential determining step (PDS) energy for POD reactions, with the diCe6c@Cu-sub site exhibiting the lowest energy barrier (0.970 eV). Simultaneously, Cu single sites suppress HORAC activity by raising PDS energies at most sites. This dual effect of enhancing ROS generation while inhibiting its elimination highlights the critical role of Cu in boosting the catalytic efficiency of Cu-CeO_2_ systems (Figure 3j).

The exploration of nanozyme design has expanded significantly, focusing on three key strategies to optimize catalytic and antibacterial performance: coordination engineering and spatial configuration optimization, doping-induced photothermal enhancements, and the integration of metal oxide supports. These approaches not only deepen our understanding of catalytic mechanisms but also pave the way for practical applications.

### 3.2. Cu Clusters

Metal nanoclusters (NCs) have become an important material for the design and development of efficient dual-catalytic-site catalysts due to their rapid development in chemical catalysis. Metal clusters have excellent chemical purity, well-defined crystal structures, high specific surface areas, and uniformly distributed catalytic sites, while facilitating the achievement of targeted spot modifications, which contribute to the establishment of clear structure–property relationships [103,104,105,106]. However, although copper has attracted much attention due to its economy, the stability of Cu NCs is inferior to that of Au NCs and Ag NCs, which limits their development. How to effectively improve the stability of Cu NCs remains an urgent challenge [104,107,108,109].

**Figure 4:**
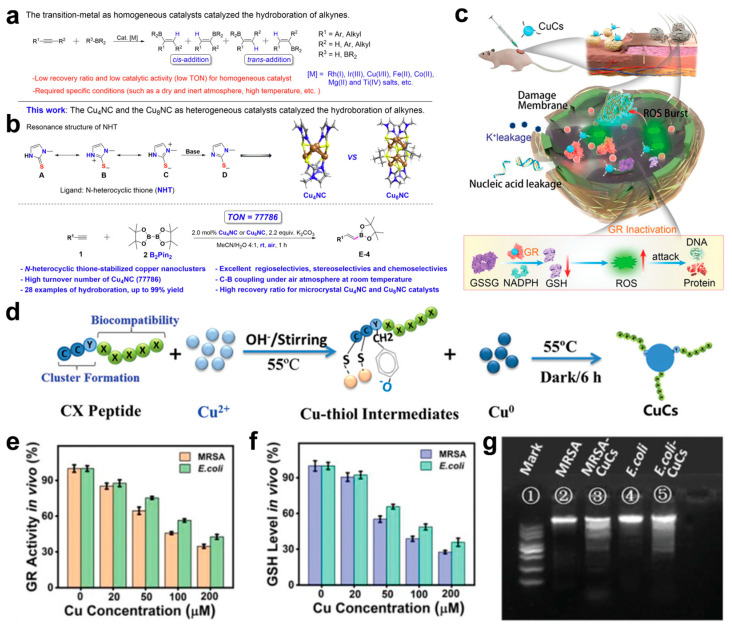
(**a**) Transition metal-catalyzed hydroboration of alkynes. (**b**) This work: microcrystalline Cu_4_NC and Cu_8_NC, as heterogeneous catalysts, catalyzed the hydroboration of alkynes. R1 represents the type of functional group. (**c**) Schematic illustration of CuCs healing local MRSA skin wound infection. The arrows represent the changing trend. (**d**) Formation of CuCs. (**e**) Enzymatic activity of GR in MRSA and *E. coli* after treatment with CuCs. (**f**) GSH levels in MRSA and *E. coli* after treatment with CuCs. (**g**) CuCs induced DNA fragmentation: (1) DNA marker; (2) genomic DNA of untreated MRSA; (3) genomic DNA of MRSA treated with CuCs; (4) genomic DNA of untreated *E. coli*; and (5) genomic DNA of *E. coli* treated with CuCs. (**a**,**b**) Reproduced with permission [110]. Copyright 2024, Nature Publishing Group. (**c**–**g**) Reproduced with permission [117]. Copyright 2021, WILEY-VCH.

Jia et al. developed a copper nanocluster (Cu NC) catalyst with a dynamic ligand effect, characterized by high stability, atomic precision, and dynamic dual catalytic sites [110]. The designed microcrystalline Cu_4_NCs feature well-defined structures and demonstrate outstanding catalytic performance in the borohydride reaction of alkynes (Figure 4a), achieving superior regioselectivity, stereoselectivity, and 100% chemoselectivity. Under mild conditions (an air atmosphere, proton solvent, and room temperature), the reaction yield reached up to 99%. Notably, the catalyst achieved an exceptional turnover number (TON) of 77,786, significantly surpassing other reported catalysts (Figure 4b).

The size of metal nanoclusters (NCs) is between that of small molecules and nanoparticles, combining the unique properties of molecules and nanomaterials. As functional building blocks, metal NCs can be assembled into materials with multifunctional properties such as catalysis [111,112,113], luminescence [114], and imaging [115,116], which are widely used in the fields of catalysis, luminescence, and sensing.

Theanine peptides facilitate the efficient assembly of copper clusters (CuCs) through coordination interactions between their amino acid sequences and copper ions [117]. The peptide chain provides a stable chemical environment, promoting the aggregation and self-assembly of CuCs into nanostructures with small particle sizes and high surface activity. Serving as both a template and stabilizer, theanine peptides endow CuCs with excellent structural stability and biological activity (Figure 4c,d). The antibacterial mechanism behind CuCs involves two primary pathways. First, CuCs disrupt the bacterial cell wall and membrane structure, causing the leakage of intracellular components. Second, CuCs bind to glutathione reductase (GR) (Figure 4e), inhibiting its catalytic activity in reducing oxidized glutathione (GSSG) to reduced glutathione (GSH), thereby impairing the bacterial antioxidant defense system (Figure 4f). This leads to a significant accumulation of endogenous reactive oxygen species (ROS), which induce DNA fragmentation via genomic damage and accelerate cell wall rupture (Figure 4g), ultimately causing bacterial death. Importantly, the low toxicity of CuCs highlights their potential as a safe and effective strategy for combating multi-drug-resistant bacterial infections.

Cu nanoclusters have shown great application value in catalysis due to their well-defined structure, excellent catalytic performance, and versatility. In order to solve the problem of the insufficient stability of copper nanoclusters, strategies such as the dynamic ligand effect and peptide chain templates have been studied to significantly improve the stability and catalytic activity of copper nanoclusters and achieve high selectivity, high yield, and low-toxicity antibacterial properties, providing a new direction for catalysis and antibacterial applications.

### 3.3. Copper Oxides and Chalcogenides

Copper oxides and chalcogenides, composed of copper and chalcogen elements such as sulfur, selenium, and tellurium, demonstrate significant antibacterial properties due to their multifunctional mechanisms and broad adaptability. Their controlled and sustained release of Cu^2+^ ions effectively disrupts bacterial membranes and inhibits microbial metabolism, ensuring prolonged antibacterial activity [118,119,120,121]. Moreover, these materials exhibit remarkable stability under diverse environmental conditions, including extreme pH, maintaining consistent antibacterial efficacy [120,122].

**Figure 5:**
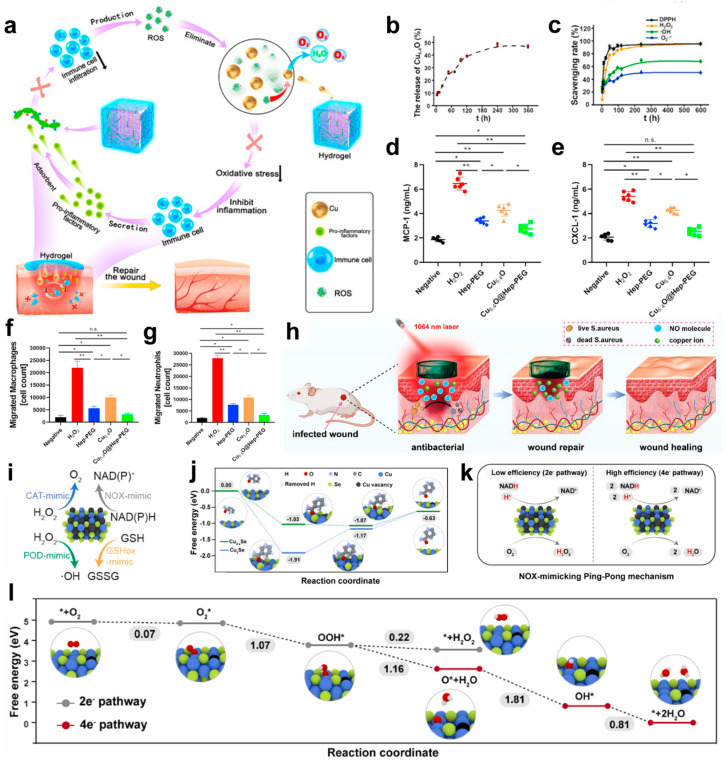
(**a**) Schematic of Cu_5.4_O@Hep-PEG hydrogels in the treatment of diabetic wounds. The downward arrow indicates a decline. (**b**) In vitro cumulative release of Cu_5.4_O USNPs from Cu_5.4_O@Hep-PEG over time. (**c**) ROS levels in untreated and Hep-PEG-, Cu_5.4_O-, and Cu_5.4_O@Hep-PEG-treated 3T3 cells incubated with 250 μM H_2_O_2_. (**d**) Concentrations of MCP-1 and (**e**) CXCL-1 incubated with 100 μM H_2_O_2_ under different treatment conditions. (**f**) The number of macrophages and (**g**) neutrophils in the lower chamber of a transwell incubated with conditioned medium collected after incubation with 100 μM H_2_O_2_ under different treatment conditions. (* *p* < 0.05; ** *p* < 0.01; n.s.: no significance, One-way ANOVA). (**h**) Synergistic treatment with mild light-induced heat, copper ions, and NO from CuS/NO Gel for infected wound healing. (**i**) Schematic illustration of multienzyme mimetic activities in Cu_2−x_Se nanozymes. (**j**) Free energy diagrams of Cu_2_Se NPs and Cu_2−x_Se nanozymes during the NADH dehydrogenation process. (**k**) Schematic illustration of the NOX mimicking Ping-Pong mechanism on Cu_2−x_Se nanozymes. (**l**) Free energy diagrams of Cu_2−x_Se nanozymes for 2e^−^ and 4e^−^ ORR pathways. (**a**–**g**) Reproduced with permission [123]. Copyright 2021, Elsevier Ltd. (**h**) Reproduced with permission [127]. Copyright 2023, WILEY-VCH. (**i**–**l**) Reproduced with permission [128]. Copyright 2024, Elsevier Ltd.

Building on these properties, Peng et al. developed Cu_5.4_O@Hep-PEG hydrogels, a multifunctional wound dressing that incorporates amine-functionalized star-shaped polyethylene glycol (starPEG) and heparin with Cu_5.4_O ultrasmall nanozymes (Figure 5a) [123]. These hydrogels demonstrated a controlled release of approximately 16% Cu_5.4_O USNPs over 24 h in PBS, highlighting their sustained release capabilities (Figure 5b). This innovative biomaterial addresses key challenges in chronic wound healing by adsorbing pro-inflammatory chemokines such as MCP-1 and IL-8, while simultaneously scavenging reactive oxygen species (ROS) (Figure 5c). Through this dual mechanism, Cu_5.4_O@Hep-PEG hydrogels effectively disrupt the inflammatory feedback loop, reduce oxidative stress, and create a balanced microenvironment conducive to wound repair and regeneration (Figure 5d–g).

In addition to ion release, copper chalcogenides, such as copper sulfide (CuS), display unique photothermal and photocatalytic properties that further enhance their antibacterial efficiency. Under near-infrared (NIR) light irradiation, CuS nanoparticles absorb light and convert it into localized heat, effectively killing bacteria without causing excessive thermal damage to surrounding tissues [124,125,126]. Leveraging these distinctive properties, the CuS/NO hydrogel represents a breakthrough in wound care by integrating photothermal therapy, sustained Cu^2+^ release, and controlled nitric oxide (NO) delivery into a single platform (Figure 5h) [127]. This multifunctional hydrogel combines mild photothermal heating and dual-phase NO release to deliver enhanced antibacterial effects while simultaneously promoting tissue regeneration through angiogenesis and collagen deposition. As a result, the CuS/NO hydrogel offers a biocompatible, efficient, and versatile solution for treating infected wounds, addressing both infection control and wound healing in a single system. Simultaneously, the photocatalytic generation of reactive oxygen species (ROS) under light exposure provides an additional mechanism for microbial eradication. These complementary effects enable copper chalcogenides to deliver broad-spectrum antibacterial activity with high efficiency.

Beyond these functionalities, defect engineering introduces further enhancements to copper-based materials by altering their electronic structures. For instance, copper defects (V_Cu_) in compounds like Cu_2−x_Se create metal-defect active centers that improve catalytic performance [128]. These centers enhance the adsorption and activation of NADH, thereby boosting dehydrogenation activity (Figure 5i). Furthermore, defect-engineered copper nanomaterials favor the efficient four-electron oxygen reduction pathway, directly converting oxygen to H_2_O rather than forming H_2_O_2_ via the less efficient two-electron pathway. This pathway significantly improves oxygen utilization and overall catalytic efficiency (Figure 5j,k). Theoretical studies further highlight that defect engineering accelerates electron transfer, enhancing both NADH oxidation and oxygen reduction, even under hypoxic conditions commonly present in infection sites or tumor microenvironments (Figure 5l). These advances in defect-engineered copper nanomaterials underline their superior catalytic activity, establishing them as highly effective agents in antibacterial and therapeutic applications.

### 3.4. Copper Alloy

The antimicrobial effect of metal alloys is mainly achieved through a threefold synergistic mechanism: metal ion release [129,130,131], surface contact sterilization [132,133,134], and corrosion microenvironment regulation [135,136,137,138,139]. The metal ions represented by silver, copper, and zinc ions disrupt the bacterial membrane structure and interfere with metabolism by inducing reactive oxygen species, while the surface metal particles directly inhibit the formation of biofilms, and the ions released during the corrosion process and the change in local pH further inhibit the proliferation of microorganisms. Among them, copper alloys outperform copper oxides, sulfides, and pure copper in terms of antibacterial properties, stability, and mechanical performance [140,141]. Through alloying, the antibacterial efficiency of copper is enhanced by optimizing ion release, increasing oxidation resistance, and improving durability, while maintaining superior mechanical properties and conductivity [142,143]. This synergistic optimization of antimicrobial efficacy with mechanical properties and corrosion resistance makes copper alloys valuable for applications in the fields of infection prevention and the control of medical devices and the development of environmentally friendly materials [144,145,146,147].

The Ti-6Al-4V-xCu alloy exemplifies this advancement, achieving superior mechanical strength, corrosion resistance, antibacterial efficacy, and biocompatibility through microstructural refinement and laser powder bed fusion (L-PBF) manufacturing [148]. The addition of copper refines the microstructure by forming a Cu-rich β phase and Ti_2_Cu precipitates, enhancing strength through solid solution and precipitation hardening while improving wear resistance. Increased copper content boosts corrosion resistance by forming a stable oxide film and reducing galvanic corrosion, aided by L-PBF’s rapid solidification process. Antibacterial properties are achieved via controlled Cu^2+^ ion release and ROS generation, while the optimized copper content ensures biocompatibility, promoting cell adhesion, osteogenesis, and angiogenesis. These features make Ti-6Al-4V-xCu alloys ideal for durable, antibacterial biomedical implants.

## 4. Looking Ahead

Copper nanomaterials have shown remarkable potential in dealing with antibiotic-resistant bacteria but still face some challenges, and further optimization and improvement are urgently needed in the future.

However, their clinical translation still faces systemic challenges. In terms of biosafety, although surface modification techniques (e.g., polydopamine coating and polyethylene glycol functionalization) can reduce the acute toxicity of the materials to mammalian cells, the dynamics of the modified layer in complex physiological environments have not yet been clarified. This “biocompatible camouflage” may interfere with the normal clearance function of macrophages, leading to the abnormal accumulation of nanomaterials in the liver and spleen organs. More importantly, it is questionable whether this simplified paradigm can truly reflect the complex immune response network in the human body, given that existing studies have generally used in vitro cellular models to assess toxicity.

The risk of drug resistance evolution needs to be revisited. Despite the multi-targeted action profile of copper ions, the adaptive evolution of bacteria through population-sensing regulation may generate cross-resistance. In addition, the oxidative stress environment triggered by copper nanomaterials may selectively promote the formation of resistant bacterial subpopulations, and these metabolically dormant bacteria establish a temporary barrier to resistance by, for example, altering membrane permeability. What is more serious is that the current drug resistance research still sticks to the detection model of “a single strain and static concentration”, completely ignoring real evolutionary scenarios such as multi-species competition and gene-level transfer in the clinical environment. This kind of experimental design, which is detached from ecological reality, may lead to a serious underestimation of the drug resistance risk of copper nanomaterials.

At the clinical translational level, current studies have fallen into the misconception of “laboratory perfectionism”. Structural control problems in large-scale production have led to batch-to-batch fluctuations in the antimicrobial effects of nanomaterials, and such instability can lead to disastrous consequences in medical applications. A more fundamental contradiction is that the existing medical regulatory system lacks dynamic safety assessment standards for nanomaterials—the long-term biological effects of nanomaterials remain uncharted as the materials undergo evolution in the body, including protein crown formation, ion release, and morphological transformation.

## 5. Summary

Overall, the growing global drug resistance crisis has created an urgent need for the development of novel antimicrobial strategies that break through the existing therapeutic paradigm. Copper-based nanomaterials, with their multimodal synergistic mechanisms, such as chemical-dynamic therapy (CDT), photothermal therapy (PTT), and photodynamic therapy (PDT), can effectively address the problem of antibiotic resistance and are expected to demonstrate the advantage of spatiotemporally controllable targeted sterilization. The structure–function modulation principle, including key factors such as electronic interactions, size effects, and single-atom doping, provides theoretical support for the precise design of antimicrobial materials. The multidisciplinary integration of copper nanomaterials not only promotes the translation of basic research to the clinic but also provides an innovative interdisciplinary reference program for the global anti-drug-resistance crisis.

## Figures and Tables

**Table 1:** Studies on the application of nanomaterials against drug-resistant bacteria in recent years.

Nanozymes	Antimicrobial Mechanism	Pathogen	Ref.
(WA)_3_GQA8C	Membrane disruption	MRSA	[17]
FeOMo_6_@WOx	Generation of ROS	MRSA	[18]
VMSNT	Membrane disruption	MRSA	[19]
In_2_S_3_/MBene	Generation of ROS	MRSA	[20]
BBR/CGA	Membrane disruption	*S. aureus* MRSA	[21]
DMAE	DNA damage	MRSA	[22]
CuGA-VAN	Disruption to bacterial signaling	*E.coli* MRSA	[15]
Ga_2_O_3_ NPs	Generation of ROS	MDR *E. coli* MDR *S. aureus*	[23]
Hb-Naf@RBCM NPs	Generation of ROS	MRSA	[24]
Au_44_(MBA)_18_	Membrane disruption DNA damage	VRE	[25]
F3FT N3FT	Membrane disruption Generation of ROS	Clinically resistant bacteria	[26]
NCefoTs	Membrane disruption Inhibition of enzyme activity	MDR *E. coli*	[27]
E–Au NPs	Membrane disruption Generation of ROS	MRSA	[28]
ZnBq/Ce6 @ZIF-8@OMV	Membrane disruption	*A. baumannii*	[29]
CAMO	Inhibition of enzyme activity	*H. pylori*	[30]
AP@Bi_2_S_3_	Membrane disruption Interference with bacterial metabolism	*E. coli* MRSA	[31]
Man-Zn^2+^/Van NPs	Membrane disruption Interference with bacterial metabolism	MRSA	[32]

## Data Availability

No new data were created or analyzed in this study.
